# Supplementary material for: Factors associated with hemolysis during extracorporeal membrane oxygenation (ECMO)—Comparison of VA- versus VV ECMO
Source: PLoS One. 2020 Jan 27;15(1):e0227793. doi: 10.1371/journal.pone.0227793 (PMC6984694; doi:10.1371/journal.pone.0227793)
Supplement: S1 Table — (DOCX) [file pone.0227793.s001.docx]

S 1 Table: Effect of 15 Fr and 17 Fr inflow cannula during VA ECMO with ECPR on hemolysis

|  | **15 Fr** | **17 Fr** | **p-value** |
| --- | --- | --- | --- |
| **Patients [n]** | 160 | 106 | - |
| **fHb, 1^st^ day [mg/l]** | 85 (55-160) | 76 (51-218) | p=0.853 |
| **fHb, 2^nd^ day [mg/l]** | 56 (43-88) | 59 (46-98) | p=0.729 |
| **p-value, 1^st^ vs. 2^nd^ day** | p≤0.001 | p=0.003 |  |
| **BF, 1^st^ day [l/min]** | 2.9 (2.2-3.3) | 3.2 (2.8-3.8) | p≤0.001 |
| **BF, 2^nd^ day [l/min]** | 2.7 (2.3-3.4) | 3.0 (2.2-3.8) | p=0.200 |
| **p-value, 1^st^ vs. 2^nd^ day** | p=0.004 | p=0.006 |  |
| **FV, 1^st^ day [cm/s]** | 20.1 (15.2-22.8) | 17.3 (15.1-21.1) | P=0.005 |
| **FV, 2^nd^ day [cm/s]** | 18.7 (15.9-23.5) | 16.5 (12.2-20.4) | P=0.005 |
| **p-value, 1^st^ vs. 2^nd^ day** | p=0.003 | p=0.006 |  |
| **ECMO time [days]** | 2 (1-4) | 3 (1-4) | p=0.886 |

Data are median (interquartile range). fHb, free plasma hemoglobin; Fr, French; BF, blood flow; FV, flow velocity; ECPR, extracorporeal cardiopulmonary resuscitation. FV was calculated by dividing blood flow (Q, cm^3^/s) through the cross-sectional area of the cannula (A, cm^2^), FV = Q/A (cm/s)
