# Supplementary material for: Factors associated with hemolysis during extracorporeal membrane oxygenation (ECMO)—Comparison of VA- versus VV ECMO
Source: PLoS One. 2020 Jan 27;15(1):e0227793. doi: 10.1371/journal.pone.0227793 (PMC6984694; doi:10.1371/journal.pone.0227793)
Supplement: S2 Table — (DOCX) [file pone.0227793.s002.docx]

S 2 Table: Effect of the pump type on the frequency of a PHT

| **Pump** | **VA ECMO** | **VV ECMO** | **p-value** |
| --- | --- | --- | --- |
| **N** | 15 | 41 |  |
| **Cardiohelp [n; %]** | 7; 47 | 14; 34 | p=0.563 |
| **Rotaflow [n; %]** | 1; 7 | 4; 10 | p=0.859 |
| **DP3 [n; %]** | 2; 13 | 8; 20 | p=0.833 |
| **Revolution [n; %]** | 4; 27 | 13; 32 | p=0.974 |
| **Others [n; %]** | 1; 7 | 2; 5 | p=0.711 |

PHT: pump head thrombosis; Pumps see Table 1.
